# Supplementary material for: Active Components of Commonly Prescribed Medicines Affect Influenza A Virus–Host Cell Interaction: A Pilot Study
Source: Viruses. 2021 Aug 3;13(8):1537. doi: 10.3390/v13081537 (PMC8402715; doi:10.3390/v13081537)
Supplement: Supplementary file 1 [file viruses-13-01537-s001.zip › viruses-1313931-supplementary.pdf]

Table S1. Active compounds of selected drugs, their suppliers and catalogue numbers.

Drug	CAS	MW	Formula	Cat N	Pur., %	Supplier
17α-Ethynylestradiol	57-63-6	296	C20H24O2	E4876-100MG	≥98	Sigma Aldrich
4-Acetamidophenol	103-90-2	151	C8H9NO2	102330050	98	Acros Organics
Acetylsalicylic acid	50-78-2	180	C9H8O4	AC158180500	99	Acros Organics
Amlodipine	88150-42-9	409	C26H31ClN2O8S	CAYM14838	≥98	Cayman Chemicals
Atorvastatin	134523-03-8	559	C33H35FN2O5	CAYM10493	≥98	Cayman Chemicals
Bumetanide	28395-03-1	364	C17H20N2O5S	CAYM14630	≥98	Cayman Chemicals
Candesartan	139481-59-7	440	C24H20N6O3	sc-217825	≥98	Santa Cruz Biotechnology
Cetirizin	83881-52-1	389	C21H27Cl3N2O3	89126-50MG	≥98	Sigma Aldrich
Cyanocobalamin	68-19-9	1355	C63H88CoN14O14P	DRE-C11798500		LGC Standards
Desloratadine	100643-71-8	311	C19H19ClN2	CAYM16931	≥98	Cayman Chemicals
Desogestrel	54024-22-5	310	C22H30O	CAYM23651	≥95	Cayman Chemicals
D-Pantothenic acid	79-83-4	219	C9H17NO5	HY-B0430	≥98	MedChemExpress
Drospirenone	67392-87-104	367	C24H30O3	CAYM23347	≥98	Cayman Chemicals
Enalapril	75847-73-3	376	C20H28N2O5	J60750.03	≥97	Alfa Aesar
Escitalopram	128196-01-0	324	C20H21FN2O	CAYM22405	≥98	Cayman Chemicals
Esomeprazole	161973-10-0	767	C34H42MgN6O9S2	CAYM17326	≥95	Cayman Chemicals
Etonogestrel	54048-10-1	324	C22H28O2	CAYM21062	≥98	Cayman Chemicals
Fluticasone propionate	80474-14-2	445	C25H31F3O5S	462101000	≥96	Acros Organics
Folic acid	59-30-3	441	C19H19N7O6	J62937.06	≥97	Alfa Aesar
Furosemide	54-31-9	331	C12H10ClN2O5S	448970010	≥97	Acros Organics
Hydroxocobalamin	13422-5 51-0	1346	C62H89CoN13O15P	CAYM24099	≥95	Cayman Chemicals
**Drug**	**CAS**	**MW**	**Formula**	**Cat N**	**Purity, %**	**Supplier**
Insulin aspart	116094-23-6	5826	C256H387N65O79S6	EPY0000349		LGC Standards
Lercanidipine	132866-11-6	612	C36H41N3O6	HY-B0612A	98.5	MedChemExpress
Levonorgestrel	797-63-7	312	C21H28O2	CAYM10006	≥95	Cayman Chemicals
Levothyroxine	25416-653	817	C15H12I4NNaO5	FT48192	≥97	Carbosynth
Losartan	114798-26-4	423	C22H23ClN6O	FL39656	≥97	Carbosynth
Metformin	1115-70-4	166	C4H12ClN5	sc-202000	≥99	Santa Cruz Biotechnology
Metoprolol	51384-51-1	267	C15H25NO3	sc-264643	97	Santa Cruz Biotechnology
Mometasone furoate	83919-23-7	521	C27H30Cl2O6	CAYM21365	≥98	Cayman Chemicals
Naproxen	22204-53-1	230	C14H14O3	CAYM70290	≥99	Cayman Chemicals
Nicotinic acid	59-67-6	123	C6H5NO2/HOOC5H4N	128290050	99.5	Acros Organics
Nifedipine	21829-25-4	346	C17H18N2O6	CAYM11106	≥98	Cayman Chemicals
Pantoprazole	102625-70-7	383	C16H15F2N3O4S	CAYM21345	≥98	Cayman Chemicals
Ramipril	87333-19-5	417	C23H32N2O5	FC27676	≥98	Cymit Quimica
Riboflavin	83-88-5	376	C17H20N4NaO9P	A11764.14	98	Alfa Aesar
Salbutamol	18559-94-9	239	C13H21NO3	CAYM21003	≥98	Cayman Chemicals
Salmeterol	89365-50-4	416	C25H37NO4	HY-14302	99.7	MedChemExpress
Sertraline	79559-97-0	306	C17H18Cl3N	462190010	≥98	Acros Organics
Simvastatin	79902-63-9	419	C25H38O5	458840010	98	Acros Organics
Tamsulosin	106463-17-6	445	C20H29ClN2O5S	CAYM24020	≥98	Cayman Chemicals

Thiamine	67-03-8	337	HC12H17ON4SCl2	148990100	99	Acros Organics
Valsartan	137862-53-4	436	C24H29N5O3	sc-220362	≥98	Santa Cruz Biotechnology
Venlafaxine	99300-78-4	277	C17H27NO2	HY-B0196A	98	MedChemExpress
Vitamin D2	50-14-6	397	C28H44O	CAYM11791	≥98	Cayman Chemicals
Vitamin D3	67-97-0	385	C27H44O	CAYM11792	≥98	Cayman Chemicals

Table S2. The most dispensed medicines in Central Norway in 2019 sorted by daily defined dosage (DDD).

ATC	Active compounds	Indications	DDD
C10AA05	Atorvastatin	Hypercholesterolemia	24 480 740
B01AC06	Acetylsalicylic acid	Pain, fever or inflammation	16 113 802
C08CA01	Amlodipine	Hypertension and coronary artery disease	10 990 638
C09CA06	Candesartan	Hypertension	9 404 137
A02BC02	Pantoprazole	Erosive esophagitis and Zollinger-Ellison syndrome	8 626 254
R06AE07	Cetirizine	Hay fever, allergies, angioedema, and urticaria	8 377 836
C09AA05	Ramipril	Hypertension and congestive heart failure	8 034 213
N02BE01	Paracetamol	Pain and fever	7 548 361
C10AA01	Simvastatin	Hypercholesterolemia	7 117 959
H03AA01	Levothyroxine sodium	Thyroid hormone deficiency	6 701 185
G03AA07	Levonorgestrel	Birth control (in combination with the estrogen ethinylestradiol), emergency birth control	6 677 552
G03AA07	Ethinylestradiol	Birth control and treatment of menopausal symptoms in combination with progestins	6 677 552
B03BB01	Folic acid	Folate deficiency	6 598 841
A12AX	Vitamin D2	Vitamin D deficiency	5 675 283
A11CC05	Vitamin D3 (colecalciferol)	Vitamin D deficiency	5 332 710
R06AX27	Desloratadine	Allergic rhinitis, nasal congestion	5 309 712
C07AB02	Metoprolol	Hypertension and coronary artery disease	5 264 848
A02BC05	Esomeprazole	Gastroesophageal reflux disease, erosive esophagitis, duodenal ulcers	5 152 910
N06AB10	Escitalopram	Depression, generalized anxiety disorder	4 728 187
A10BA02	Metformin	Type 2 diabetes, polycystic ovary syndrome	4 351 201
G03AC08	Etonogestrel	Birth control	4 276 000
M01AE52	Naproxen	Pain and fever caused by inflammation	4 031 853
C09CA03	Valsartan	Hypertension and congestive heart failure	3 514 084
C09CA01	Losartan	Hypertension	3 491 802
G03AC09	Desogestrel	Birth control and menopausal symptoms	3 301 519
B03BA03	Hydroxocobalamin	Vitamin B12 deficiency	3 182 950
R03AC02	Salbutamol	Asthma	3 018 486
A11E	D-Pantothenic acid*	Vitamin B deficiency	2 872 185

A11E	Thiamine*	Vitamin B deficiency	2 872 185
A11E	Riboflavin*	Vitamin B deficiency	2 872 185
A11E	Nicotinic acid*	Vitamin B deficiency	2 872 185
C03CA01	Furosemide	Hypertension and edema	2 864 836
R03AK06	Fluticasone propionate	Asthma, allergic rhinitis, atopic dermatitis	2 668 274
C08CA13	Lercanidipine	Hypertension	2 667 274
B03BA01	Cyanocobalamin	Vitamin B12 deficiency	2 406 614
C03CA02	Bumetanide	Heart failure	2 354 487
N06AB06	Sertraline	Depression	2 129 315
A10AB05 and A10AD05	Insulin aspart	Diabetes mellitus type 1 and 2	2 101 161
G04CA02	Tamsulosin	Benign prostatic hyperplasia, kidney stones, acute urinary retention	2 065 873
R01AD09	Mometasone furoate	Symptoms in nose caused by allergy or polyps	1 968 160
N06AX16	Venlafaxine	Depression, general anxiety disorder	1 840 761
R03AK06	Salmeterol	Asthma	1 833 358
C08CA05	Nifedipine	Hypertension and angina pectoris	1 773 040
C09AA02	Enalapril	Hypertension, diabetic kidney disease and heart failure	1 770 279
G03AA12	Drospirenone	Birth control and menopausal symptoms	1 725 864

** B-Tonin, TroBe and nycoplus B-kompleks include d-pantothenic acid (vitamin B5), thiamine (vitamin B1), riboflavin (vitamin B2) or nicotinic acid (vitamin B3).*
